# Autosomal recessive MALTA syndrome with compound heterozygous *MYH9* variants: Structural modeling reveals disrupted myosin-9 assembly

**DOI:** 10.1016/j.xjidi.2026.100507

**Published:** 2026-07-09

**Authors:** Etta Hanlon, Katherine L. Brown, Haley M. Michel, Clinton Roby, Matthew G. Urbano, Dzenis Mahmutovic, Priyenka Khatiwada, Daniel G.S. Capelluto, Jane Gay, Anne M. Brown, Douglas J. Grider, Carla V. Finkielstein

**Affiliations:** 1 Fralin Biomedical Research Institute, Virginia Tech Carilion, Roanoke, Virginia, USA; 2 Virginia Tech Carilion School of Medicine, Roanoke, Virginia, USA; 3 Department of Biochemistry, Virginia Tech, Blacksburg, Virginia, USA; 4 Department of Biological Sciences, Virginia Tech, Blacksburg, Virginia, USA; 5 Fralin Life Sciences Institute, Virginia Tech, Blacksburg, Virginia, USA; 6 Section of Dermatology, Department of Internal Medicine, Virginia Tech Carilion School of Medicine, Roanoke, Virginia, USA; 7 University Libraries, Virginia Tech, Blacksburg, Virginia, USA; 8 Department of Basic Science Education, Virginia Tech Carilion School of Medicine, Roanoke, Virginia, USA; 9 Dominion Pathology Associates, Roanoke, Virginia, USA; 10 Department of Surgery, Virginia Tech Carilion School of Medicine, Roanoke, Virginia, USA

**Keywords:** Autosomal recessive inheritance, Elastic fiber aggregation, MALTA syndrome, Microcystic adnexal carcinoma, *MYH9*

## Abstract

Distinguishing microcystic adnexal carcinoma from MALTA (*MYH9*-associated elastin aggregation) syndrome is critical to prevent unnecessary aggressive surgery. We define a variant of MALTA histologically characterized by benign deep syringoid ductal proliferation with elastic fiber aggregation (denoted as MALTA-BDSDP). A male in his early twenties presented with bilateral facial lesions. Germline sequencing identified 3 heterozygous *MYH9* variants: c.1363G>A (p.Gly455Ser) in the motor domain and c.4490G>A (p.Arg1497Gln) and c.4876A>G (p.Ile1626Val) in the tail domain. To our knowledge, Gly455Ser and Arg1497Gln represent previously unreported variants that show Mendelian segregation in this family. The proband inherited Gly455Ser and Ile1626Val in cis from one parent and Arg1497Gln from the other, resulting in compound heterozygosity. His sibling carried identical variants but remained asymptomatic, demonstrating incomplete penetrance. Molecular modeling predicted that these sequence variants destabilize Myosin-9 through disrupted coiled–coil interactions and altered local packing. Uniquely, compound heterozygosity in the affected sibling suggests an autosomal recessive inheritance pattern, contrasting with previously reported dominant MALTA cases and expanding the known genetic spectrum. Histopathology revealed preserved myoepithelial architecture, an absence of perineural invasion, and pathognomonic elastic fiber aggregates, features distinguishing MALTA-BDSDP from microcystic adnexal carcinoma. These findings prevent misdiagnosis and enable appropriate genetic counseling.

## Graphical abstract

**Figure:**
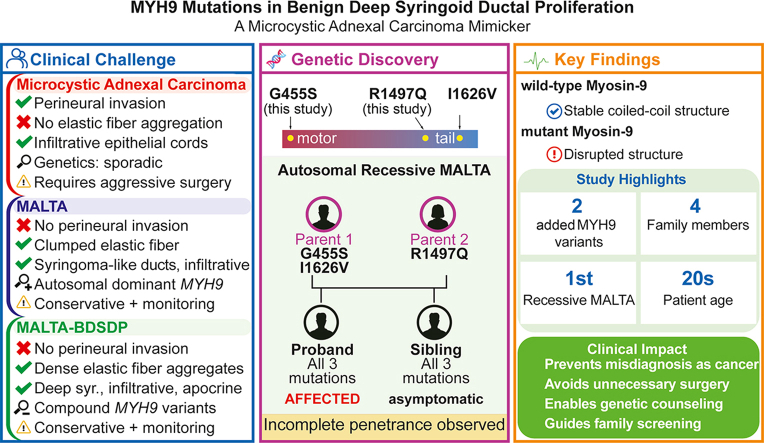

## Introduction

Microcystic adnexal carcinoma (MAC) is a rare, locally aggressive sweat gland tumor that poses a significant diagnostic challenge owing to its histologic overlap with benign adnexal lesions (Huang et al, 2023). MAC typically presents as a firm nodule on the head or neck with deep dermal and subcutaneous infiltration, keratin-filled cysts, and perineural invasion (Hoang et al, 2008). Treatment requires wide local excision with Mohs micrographic surgery, which can be disfiguring and cause functional morbidity, particularly for facial lesions. Accurate differentiation from benign mimickers is therefore critical to prevent unnecessary aggressive surgical interventions.

Several benign conditions can mimic MAC histologically, including syringomas and rare inherited syndromes (Fewings et al, 2019; Hoang et al, 2008; Schaller et al, 2010). In 2019, Fewings et al (2019) unified 2 inherited conditions—Nicolau-Balus syndrome and Rombo syndrome—as MALTA (*MYH9*-associated elastin aggregation) syndrome, characterized by benign ductal proliferations resembling MAC and abnormal elastic fiber deposition in the dermis (Fewings et al, 2019; Schaller et al, 2010). Unlike MAC, MAC-like ductal proliferations occurring in MALTA syndrome lack perineural invasion and preserve myoepithelial architecture, which are critical distinguishing features (Fewings et al, 2019; Hoang et al, 2008; Schaller et al, 2010). MALTA syndrome is caused by germline pathogenic variants in the *MYH9* gene, which encodes nonmuscle Myosin IIA (referred to as Myosin-9 in the remaining parts of this paper), a cytoskeletal motor protein essential for cellular architecture and elastic fiber homeostasis (Fewings et al, 2019; Fostier et al, 2023; Pecci et al, 2018).

To date, all reported MALTA cases have demonstrated autosomal dominant inheritance, with single heterozygous *MYH9* variants presenting in patients with normal platelets and no systemic features (Fewings et al, 2019; Fostier et al, 2023; Pecci et al, 2018). This distinguishes MALTA from other *MYH9*-related conditions, such as MATINS (macrothrombocytopenia with nephritis or hearing loss) (Fostier et al, 2023; Lalwani et al, 2000) and DFNA17 (hereditary deafness) (Lalwani et al, 2000), which involve systemic manifestations. Recent evidence links *MYH9* pathogenic variants to early-onset cutaneous malignancies, expanding the clinical relevance of these variants beyond benign dermatologic findings. An early report described a family with MALTA syndrome carrying a pathogenic variant in the *MYH9* myosin head domain that segregated with multiple early-onset cutaneous squamous cell and basal cell carcinomas (Fostier et al, 2023).

In this study, we report a patient presenting with bilateral facial lesions initially concerning for MAC but ultimately found to have what is, to our knowledge, a previously uncharacterized subtype of MALTA syndrome. This case is notable for 3 key contributions. First, we define benign deep syringoid ductal proliferation (BDSDP) with elastic fiber aggregation (denoted as MALTA-BDSDP) as a distinct histopathologic entity that requires differentiation from both classic MALTA and MAC. Second, germline sequencing identified what are, to our knowledge, 2 previously unreported *MYH9* variants (encoding for p.Gly455Ser and p.Arg1497Gln) plus p.Ile1626Val. Family segregation analysis revealed compound heterozygosity in both siblings, consistent with an autosomal recessive inheritance pattern. Third, molecular modeling predicted that these sequence variants synergistically destabilize Myosin-9 through disrupted coiled–coil interactions and altered local packing, providing mechanistic insights into genotype–phenotype correlations. This case challenges the established dominant inheritance model, expands MALTA's phenotypic spectrum, and demonstrates how integrated histopathologic–genetic analysis prevents surgical overtreatment.

## Results

### Case presentation and diagnostic challenge

**Figure 1:**
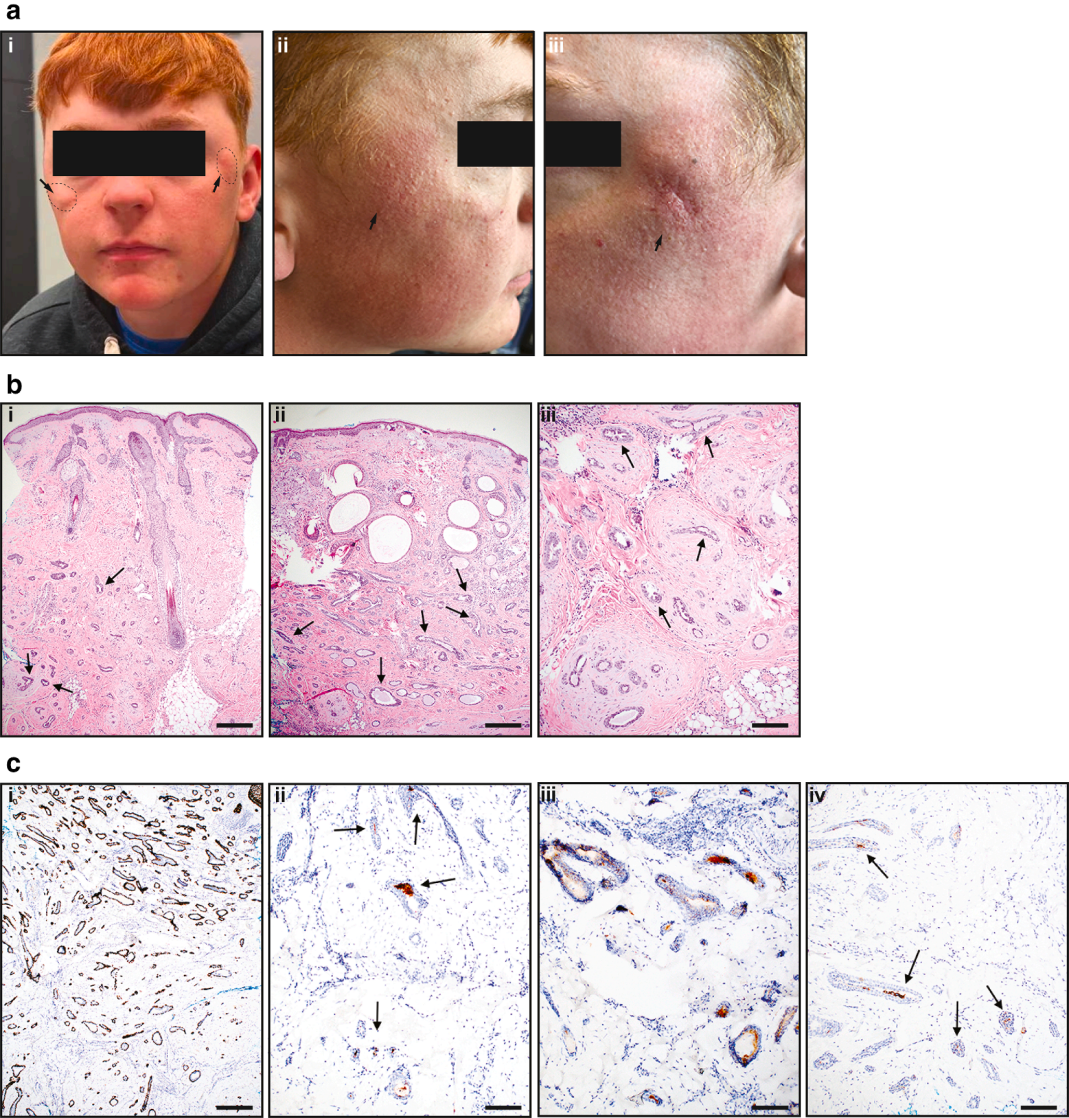
**Clinical presentation and histopathological features of a patient with MALTA syndrome with BDSDP.** (**a**) Clinical photograph (i) showing nodular erythematous swellings on the right infraorbital region (ii) and left zygomatic arch (iii), measuring approximately 2.5 and 3.2 cm in diameter, respectively. (**b**) H&E-stained tissue sections from the right infraorbital region at ×4 (i), with arrows indicating ducts exhibiting apocrine-like changes. H&E-stained tissue sections from the left zygomatic arch lesion at ×4 (ii) and ×10 (iii) reveal keratin horn cysts in the upper dermis, a feature mimicking MAC, along with myoid/collagenous stroma surrounding the cords and ductular proliferation, which are not typical of MAC. Arrows mark ducts with features of apocrine differentiation. Bar = 100 μm. (**c**) Immunohistochemical ancillary studies support apocrine differentiation: (i) p63 staining highlights the nuclei of the basal myoepithelial cell layer of the infiltrative ducts (×10); (ii) GCDFP-15 focally marks the luminal duct surface, a feature sometimes seen with apocrine differentiation (×10); (iii) EMA marks the luminal surface of the infiltrative glandular duct structures (×10); and (iv) CD15 positivity along the lumen surface and within the infiltrative ducts favors apocrine over eccrine origin (×10). Bar = 100 μm. The patient provided written informed consent for the use of his image in this publication. BDSDP, benign deep syringoid ductal proliferation; MAC, microcystic adnexal carcinoma.

A male in his early twenties presented with 2 longstanding, stable erythematous plaques: one on the right infraorbital region and another on the left zygomatic arch (Figure 1a). The bilateral facial presentation and young age immediately raised concerns for either an aggressive malignancy or an inherited condition. Comprehensive clinical evaluation revealed no milia, atrophodermia vermiculata, acral cyanosis, hypotrichosis, syringomas, trichoepitheliomas, basal cell carcinoma, or squamous cell carcinoma in the proband. Platelet count and mean platelet volume were within normal limits, and no hearing loss was reported. Family members (parents and sibling) reported no similar cutaneous features; those who consented to clinical examination showed no abnormalities. The initial clinical differential diagnosis included MAC, which would necessitate disfiguring wide local excision with Mohs micrographic surgery, versus a benign inherited adnexal proliferation requiring only conservative monitoring. This distinction was critical for determining appropriate management.

Histologic evaluation initially heightened concern for MAC, revealing keratin cysts in the upper dermis with extensive ductal proliferations extending deep into the subcutaneous tissues, features characteristic of locally aggressive sweat gland carcinoma. However, a systematic evaluation for distinguishing features revealed 3 critical findings that excluded MAC: (i) complete absence of perineural invasion across multiple tissue sections; (ii) preserved p63+ myoepithelial cell architecture surrounding all ducts; and (iii) presence of dense, ball-like elastic fiber aggregates in the papillary dermis, a pathognomonic feature not seen in MAC (Figure 1b and c).

Additional malignant entities were considered in the differential diagnosis. Low-grade adenosquamous carcinoma, a variant typically associated with breast tissue, was excluded on the basis of the patient’s sex and anatomic location (Zhang and Fong, 2021). Cutaneous adenosquamous carcinoma was ruled out by the presence of intact peripheral myoepithelial cells (Christie et al, 2025). Squamoid eccrine ductal carcinoma, although rare, was also excluded on the basis of distinctive histologic features (Svoboda et al, 2021).

### Histopathologic diagnosis: defining BDSDP

Immunohistochemical analysis demonstrated apocrine differentiation with focal GCDFP-15+ and EMA+ expression along the inner ductal surfaces and CD15+ cytoplasmic and membranous staining, confirming an apocrine rather than eccrine lineage (Figure 1c). Notably, apocrine differentiation has been previously described in a MAC-like ductal proliferation in a patient with suspected MALTA syndrome who did not undergo *MYH9* variant testing (Llamas-Velasco et al, 2019). The ductal proliferations extended unusually deep into the subcutaneous tissue (deeper than classic MALTA), with a prominent syringoid architecture and apocrine features, leading us to establish the term BDSDP with elastic fiber aggregation as a distinct histopathologic variant of MALTA syndrome.

The diagnostic criteria distinguishing BDSDP from MAC include (i) absence of perineural invasion, even in extensive infiltrative processes; (ii) preserved myoepithelial layer confirmed by p63 immunoreactivity; and (iii) pathognomonic elastic fiber aggregates in the papillary dermis. These features, combined with bilateral presentation in a young patient, establish a benign diagnosis and prompt genetic investigation for MALTA syndrome rather than proceeding with aggressive surgical intervention.

### Genetic investigation reveals compound heterozygosity

**Figure 2:**
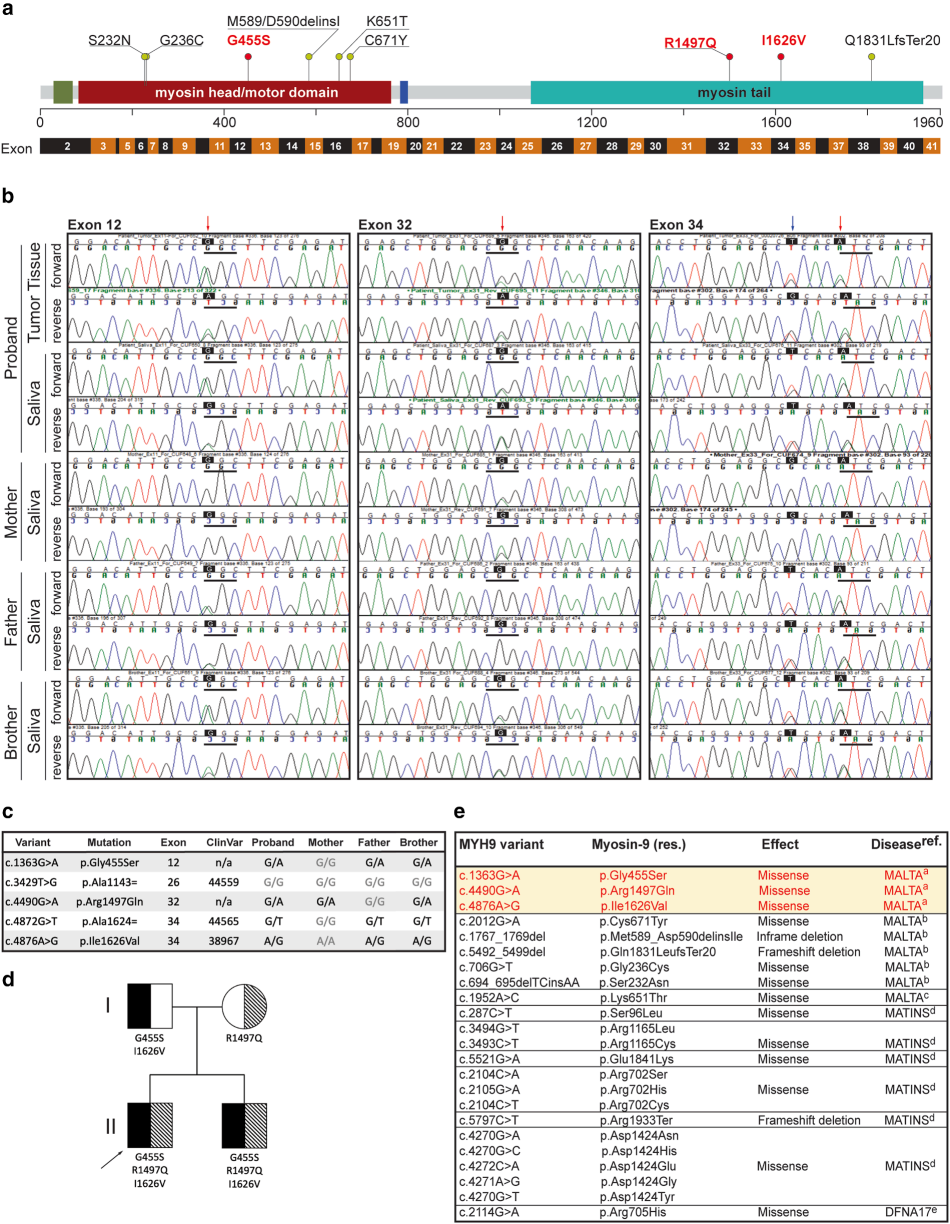
**Genomic identification of familial *MYH9* variants.** (**a**) The human *MYH9* gene (chromosome 22) transcribes a 7451 bp full-length mRNA (NM_002473), comprising 41 exons, 40 of which encode a canonical protein of 1960 amino acids known as NMHC IIA (nonmuscle myosin heavy chain II A), also referred to as Myosin-9 (UniProt P35579). The domain architecture of Myosin-9 includes an ATP-binding motif (residues 174–181); a myosin N-terminal SH3-like domain (residues 27–77, shown in green); a myosin motor domain (residues 83–764, shown in maroon); an IQ (Ile/Gln) motif (residues 783–799, shown in blue), typically found in proteins regulated by calcium/calmodulin signaling; and a myosin tail domain (residues 1067–1923, shown in cyan). Previously reported pathogenic variants (missense, deletions/insertions, and frameshifts) linked to MALTA are shown in green lollipops; red lollipops indicate newly identified variants identified in the proband. Below, a schematic of the 41 *MYH9* exons aligned with the corresponding protein regions. (**b**) Representative sequencing chromatograms of gDNA from tumor and saliva samples from the proband and immediate family members. Detected variants are shown: c.1363G>A (p.Gly455Ser; exon 12), c.4490G>A (pArg1497Gln; exon 32), and c.4876A>G (pIle1626Val; exon 34). Black boxes highlight the variant positions, red arrows denote altered peak in the chromatogram, and blue arrow indicates a synonymous variant in exon 34 (c.4872G>T; p.Ala1624=). Codon positions are marked with solid black lines. Double peaks indicate heterozygosity. The *MYH9* c.1363G>A (p.Gly455Ser), c.4490G>A (p.Arg1497Gln), and c.4876A>G (p.Ile1626Val) pathogenic variants were confirmed as heterozygous in both normal tissue and saliva, indicating that these are constitutional (germline) variants rather than somatic changes confined to the tumor. (**c**) Table summarizing the *MYH9* variants identified in gDNA from saliva samples of all individuals in the pedigree, including corresponding protein changes, exon locations, and ClinVar accession numbers (if available). For each variant, the genotype (eg, G/A, G/G, G/A, G/T, A/G) is provided for the proband and first-degree relatives. (**d**) Pedigree chart illustrating inheritance patterns of 3 *MYH9* variants across 2 generations (I and II). gDNA from saliva samples revealed that the father (I-1) carries the Gly455Ser and Ile1626Val pathogenic variants on the same allele, whereas the mother (I-2) carries the Arg1497Gln variant. The chart shows a Mendelian segregation pattern, with paternal inheritance of Gly455Ser and Ile1626Val and maternal inheritance of Arg1497Gln in both male offspring (II-1 and II-2). The proband (II-1) is indicated by an arrow. Variant distribution is shown using patterned fills: solid black for Gly455Ser and Ile1696Val and diagonal stripes for Arg1497Gln. Squares represent males; circles denote females. (**e**) Summary of *MYH9* variants associated with MALTA (*MYH9*-associated elastin aggregation), MATINS (macrothrombocytopenia and granulocyte inclusions with or without nephritis or sensorineural hearing loss), and DFNA (deafness, autosomal dominant 17) phenotypes, including those located in the coding region and reported in this work. References: (**a**) this work, (**b**) Fewings et al (2019), (**c**) Fostier et al (2023), (**d**) only included 6 hotspot pathogenic variants of 107 MATINS variants reported (Shen et al, 2024), and (**e**) Lalwani et al (2000). ATP, adenosine triphosphate; gDNA, genomic DNA.

Given the bilateral facial involvement, young age of onset, longstanding stability without malignant progression, and the presence of elastic fiber aggregates, a hallmark of MALTA syndrome, germline *MYH9* testing was pursued. Sanger sequencing of all 40 coding exons identified 3 heterozygous missense variants: c.1363G>A (p.Gly455Ser) in the motor domain and c.4490G>A (p.Arg1497Gln) and c.4876A>G (p.Ile1626Val) in the tail domain (Figure 2a). All 3 variants were confirmed as germline by their detection in both lesional tissue and saliva samples (Figure 2b and c). Critically, Gly455Ser and Arg1497Gln had not been previously reported in MALTA syndrome, variant databases, or the general population; computational pathogenicity prediction classified Gly455Ser as "pathogenic strong" (Kopanos et al, 2019). Eight additional intronic variants were identified but were predicted to have no functional impact (Table 1).

**Table 1:** Intronic *MYH9* Variants Identified in the Proband

Variant	Intron	ClinVar	SNP	Proband
NM_002473.4:c.333+59_333+62delinsAGC	2	N/A	rs372137829	Heterozygous
NM_002473.6(*MYH9*):c.1012+56C>T	9	1289525	rs3752463	Heterozygous
NM_002473.6(*MYH9*):c.1012+63G>A	9	N/A	N/A	Heterozygous
NM_002473.6(*MYH9*):c.1554+7A>G	13	44550	rs3752462	Heterozygous
NM_002473.6(*MYH9*):c.1555-71T>C	13	1243503	rs2157257	Homozygous
NM_002473.6(*MYH9*):c.1728+10G>A	14	44551	rs2413396	Homozygous
NM_002473.6(*MYH9*):3837+25C>T	28	258745	rs4821478	Heterozygous
NM_002473.6(*MYH9*):c.5061+57C>T	35	1236568	rs13053731	Heterozygous

Abbreviation: N/A, not applicable.

Family segregation analysis revealed an unexpected inheritance pattern that challenges the established MALTA genetic model. The proband inherited Gly455Ser and Ile1626Val in cis (on the same chromosome) from the father and Arg1497Gln from the mother, resulting in compound heterozygosity for 3 distinct *MYH9* variants (Figure 2d). His sibling (late teens) inherited the identical combination of all 3 variants but remained completely asymptomatic, demonstrating incomplete penetrance that may reflect age-dependent expressivity. Most significantly, both heterozygous parents (late forties) carrying single or paired variants showed no cutaneous manifestations.

This segregation pattern contrasts sharply with all previously reported MALTA cases, which demonstrated autosomal dominant inheritance, with single heterozygous pathogenic variants being sufficient to produce clinical disease (Figure 2e). The requirement for compound heterozygosity and the absence of disease in single-variant carriers indicate autosomal recessive inheritance with incomplete penetrance, which, to our knowledge, is previously unreported in MALTA syndrome. This finding fundamentally expands the genetic spectrum of *MYH9*-related cutaneous disease and has immediate implications for genetic counseling and family screening protocols.

### Molecular modeling provides mechanistic insights

**Figure 3:**
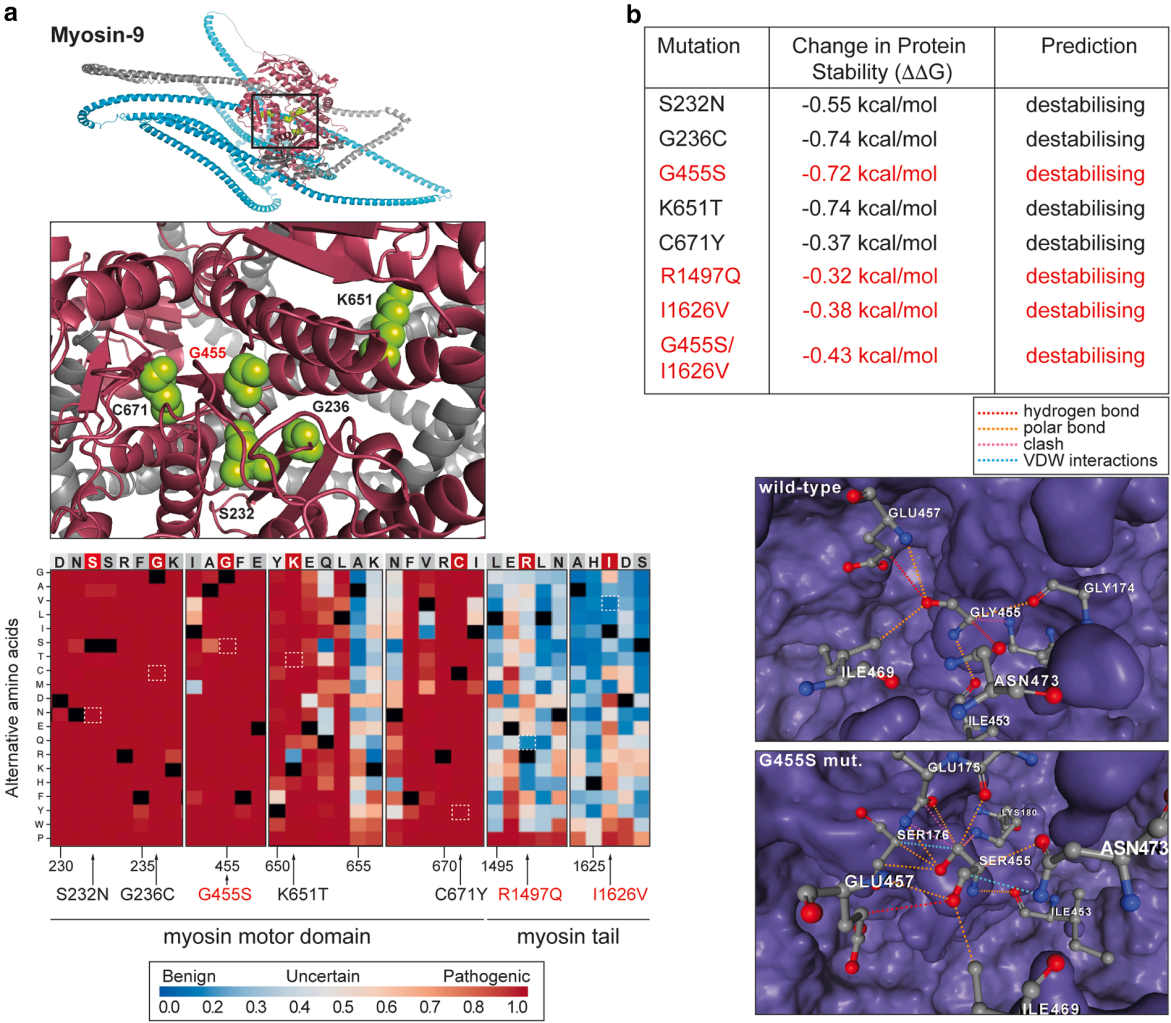
**In silico characterization of MHY9 variants.** (**a**) Cartoon representation of AlphaFold-predicted Myosin-9 structure, with domains color coded as in Figure 2a. The boxed region within the head/motor domain is shown enlarged in the middle panel. Residues previously altered in patients with MALTA syndrome are shown as green spheres. The bottom panels display the AlphaMissense pathogenicity heatmap for the amino acid region surrounding the wild-type residue (highlighted by a red box in the top sequence). The corresponding mutated substitution is indicated by a dashed white box. Unlike mutations in the tail domain that received low pathogenicity scores (Arg1497Gln: 0.126 and Ile1626Val: 0.086), residues Ser232Asn, Gly236Cys, Gly455Ser, Lys651Thr, and Cys671Tyr showed high pathogenicity scores (0.999, 0.998, 0.997, 0.983, and 1.000, respectively), supporting the interpretation that these variants likely disrupt *MYH9* function. The heatmap color gradient represents AlphaMissense pathogenicity scores, ranging from blue (low probability of pathogenicity, score near 0) to red (high probability of pathogenicity, score near 1). **(b)** DynaMut2 predictions of the thermodynamic impact of various *MYH9* sequence variants. The table shows ΔΔG values, with sequence variants identified in this study highlighted in red. The bottom panels display surface and stick models of residue interactions (hydrogen bonds, polar, and Van der Waals forces) surrounding wild-type Gly455 (top) and mutant Ser455 (bottom), revealing local structural disruptions. Notably, Ser455 but not Gly455 can form a hydrogen bond with Ser176. Additional changes in nearby interactions, including those involving Asn473 and Ile453, suggest a broader effect on the local structural environment owing to the Gly455Ser substitution.

To understand why compound heterozygosity might be required for clinical manifestation, we performed structural simulations of wild-type and variant Myosin-9 protein using AlphaFold3 structure predictions (UniProt P35579) (Jumper et al, 2021; Varadi et al, 2024). Mapping of all previously reported MALTA motor domain pathogenic variants onto the predicted 3-dimensional structure revealed clustering within a confined functional region, suggesting that these variants disrupt a critical structural domain (Figure 3a, upper and middle panels) (Jumper et al, 2021; Varadi et al, 2024).

Pathogenicity prediction using AlphaMissense assigned high scores to motor domain variants (Gly455Ser, Gly236Cys, Lys651Thr, Cys671Tyr: scores 0.997–1.000), indicating severe disruption of protein function, whereas tail domain variants (Arg1497Gln, Ile1626Val) received lower pathogenicity scores (0.126 and 0.086), suggesting more subtle effects (Figure 3a, lower panel) (Cheng et al, 2023). This differential scoring pattern suggests domain-specific functional consequences.

Protein stability modeling using DynaMut2 predicted that all 3 patient variants would destabilize the Myosin-9 structure (Rodrigues et al, 2021). Quantitative analysis revealed negative ΔΔG values for all sequence variants: Gly455Ser (−0.72 kcal/mol), Arg1497Gln (−0.32 kcal/mol), and Ile1626Val (−0.38 kcal/mol), indicating reduced structural stability (Figure 3b, upper panel) (Rodrigues et al, 2021). The Gly455Ser sequence variant in the motor domain was predicted to cause local structural rearrangements, including the formation of a new hydrogen bond with Ser176 and altered interactions with neighboring residues Gly174, Ile453, Ile469, and Asn473 (Figure 3b, lower panels). These localized changes could disrupt the motor domain's normal molecular packing and adenosine triphosphate–dependent function.

**Figure 4:**
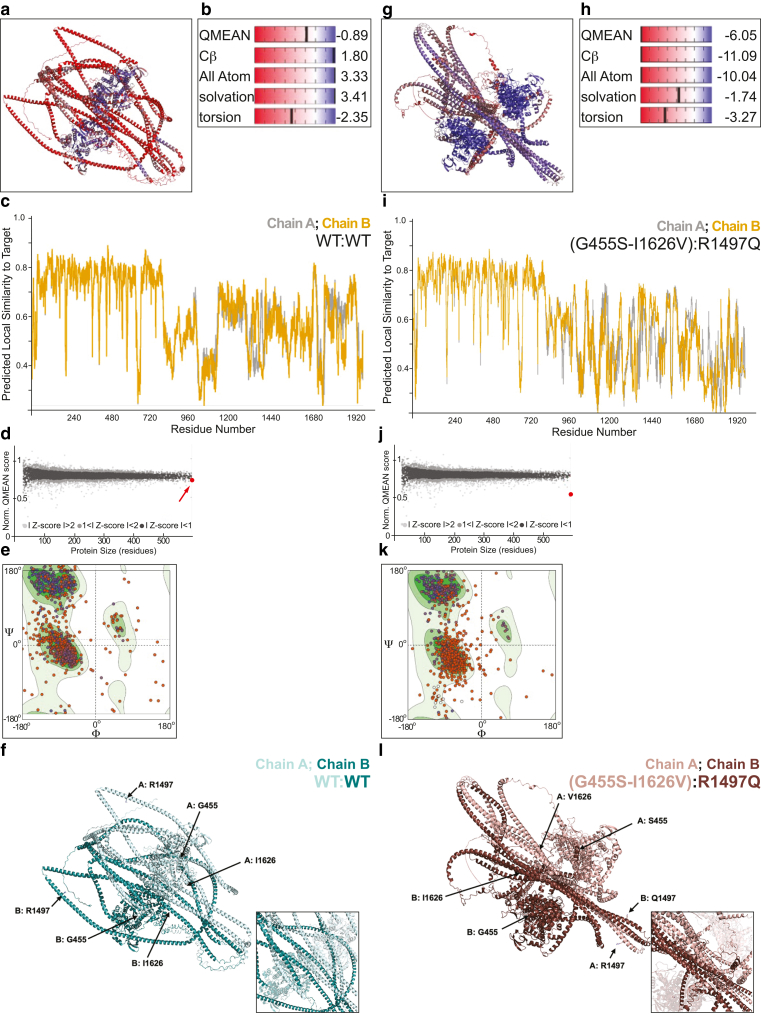
**AlphaFold-Multimer de novo model validation and quality assessment of wild-type and variant Myosin-9 dimer models.** Structures of the (**a**) wild-type and (**g**) variant Gly455S-Ile1626V:Arg1497Gln (also G455S-I1626V:R1497Q) Myosin-9 dimers, colored on the basis of the local QMEANDisCo scores. (**b**, **h**) All atom solvation torsion scores are depicted, contributing to QMEAN-based structural validation by evaluating solvent-accessible surface quality, atomic packing, and torsion angle geometry of the wild-type and variant models, respectively. (**c**, **i**) Structures of wild-type and the variant Myosin-9 are shown, with each residue colored according to local QMEANDisCo scores in chain A (gray) and chain B (gold), allowing comparison of predicted structural confidence across variants. (**d**, **j**) QMEANDisCo score provides a global and local assessment of protein model quality, with values of (**d**) 0.6 and (**j**) 0.48 for the homodimer models, respectively (indicated with a red dot). These scores are plotted against the number of residues in the homodimer, reflecting the predicted structure’s overall reliability on the basis of protein size. (**e**, **k**) Ramachandran plots illustrate the distribution of backbone dihedral angles (Φ *vs* Ψ) for individual residues, providing an assessment of sterically allowed conformations and local protein geometry quality. In plots **e** and **k**, 92.7% and 88.5% of residues, respectively, fall within favored regions (green), indicating high-quality structural modeling. (**f, l**) AlphaFold-Multimer de novo models of the (**f**) wild-type Myosin-9 homodimer and (**l**) the patient-derived mutant dimer are shown. In the wild-type model, chain A (light teal) and chain B (dark teal) include residues Gly455, Arg1497, and Ile1626. In the mutant model, residues were assigned on the basis of familial segregation: chain A (light brown) contains sequence variants Ser455 and Val1626, whereas chain B (dark brown) includes the Gln1497 variant.

**Figure 5:**
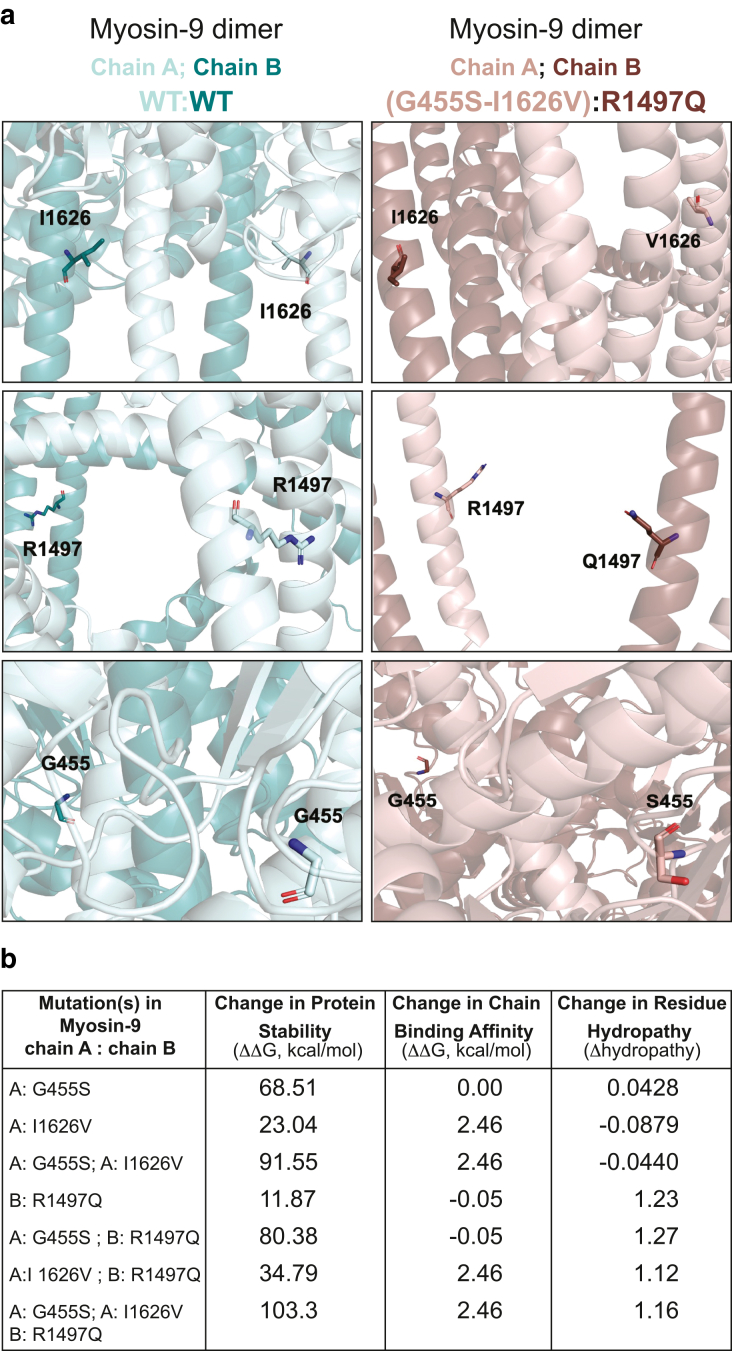
**In silico prediction of structural and thermodynamic impacts of *MYH9* variants on Myosin-9 dimer stability and binding affinity.** AlphaFold-Multimer was used to generate the predicted Myosin-9 dimer structures (chain A and chain B), including the WT and variant forms. (**a**) Myosin-9 Gly455S-Ile1626V:Arg1497Gln (also G455S-I1626V:R1497Q) sequence variants. WT (teal) and variants (brown) structures showing the interface of interactions for Ile1626 (I1626, top panels), Arg1497 (R1497, middle panels), and Gly455 (G455, bottom) are shown. Residues are shown as sticks and colored on the basis of atom type. (**b**) The table quantifies the impact of each sequence variant or their combination on key protein characteristics: Variant(s) in Myosin-9: Lists the specific amino acid changes (eg, G455S, R1497Q, I1626V) and indicates which chain (A or B) of the Myosin-9 dimer the sequence variant is located on. Change in protein stability (*ΔΔG*, kcal/mol): Represents the predicted change in the overall thermodynamic stability of the protein due to the sequence variant (kcal/mol). Positive values typically indicate destabilization, whereas negative values suggest stabilization; change in chain binding affinity (*ΔΔG*, kcal/mol): Reflects the predicted change in the binding strength between the 2 Myosin-9 dimer chains (A and B) as a result of the mutation; change in residue hydropathy (Δhydropathy): Indicates the predicted change in the hydropathy (hydrophobicity or hydrophilicity) of the mutated residue, with specific values provided. The table includes analyses for single mutations (eg, A: I1626V, A: G455S, B: R1497Q) and combinations, such as the G455S and I1626V mutations on chain A, and their occurrence with R1497Q on chain B, reflecting complex allelic configurations. WT, wild-type.

Homodimer modeling using AlphaFold-Multimer with Amber minimization revealed the potential mechanism underlying recessive inheritance. Wild-type Myosin-9 displayed an intact coiled–coil structure throughout the tail domain, which is essential for proper dimer assembly and filament formation (Figure 3c, left panel). In contrast, the compound heterozygous mutant model carrying all 3 patient variants showed marked disruption of the coiled–coil structure near the position of Ile1626Val (Figure 3c, right panel). Stability analysis using Prime MM-GBSA predicted reduced homodimer stability, with the specific combination of sequence variants found in the patient (Gly455Ser-Ile1626Val in cis paired with Arg1497Gln in trans) showing the greatest destabilization (ΔΔG = –0.43 kcal/mol for the combined effect) (Figures 3b, 4, and 5).

The Ile1626Val substitution was predicted to disrupt critical hydrophobic interactions required for maintaining coiled–coil integrity, consistent with prior biochemical studies demonstrating that isoleucine-to-valine substitutions reduce structural stability in coiled–coil proteins (Zhu et al, 1993). The combination of motor domain destabilization (Gly455Ser affecting adenosine triphosphate–dependent function and local packing) with tail domain coiled–coil disruption (Ile1626Val/Arg1497Gln impairing dimer assembly) was predicted to cause synergistic destabilization across 2 critical functional domains. This synergistic effect may explain why compound heterozygosity is required for clinical disease; single variants affecting one domain may be partially compensated by wild-type function, whereas simultaneous perturbations in both motor and tail domains exceed the functional threshold for cytoskeletal regulation and elastic fiber homeostasis.

The incomplete penetrance demonstrated by the asymptomatic sibling (late teens) carrying identical pathogenic variants indicates that additional genetic or environmental modifiers influence phenotypic expression. Longitudinal follow-up of the sibling and parents will be informative for determining whether individual variants alone can drive pathogenicity with advancing age, an important consideration for genetic counseling and risk assessment in family members.

## Discussion

This case addresses a critical clinical challenge: distinguishing benign adnexal proliferations from MAC, a distinction that determines whether patients undergo disfiguring facial surgery or conservative monitoring. We report 3 innovations that expand the understanding of MALTA syndrome while providing practical diagnostic guidance. First, we establish BDSDP as a distinct histopathologic subtype of MALTA with unusually deep subcutaneous extension and prominent apocrine features. Second, to our knowledge, we demonstrate a previously unreported case of MALTA syndrome exhibiting autosomal recessive inheritance through compound heterozygosity, challenging the established dominant model. Third, structural simulations reveal synergistic destabilization across motor and tail domains, explaining why multiple *MYH9* variants may be required for clinical pathogenic manifestation.

Accurate BDSDP–MAC differentiation has an immediate clinical impact. MAC requires wide excision with Mohs surgery, causing significant facial morbidity, whereas MALTA-BDSDP requires only monitoring. The key histopathologic and genetic features that distinguish these entities are described below. In MAC, perineural invasion is typically present, myoepithelial cells (p63+) are lost, infiltration is deep and destructive, elastic fibers appear normal, and genetics are sporadic. In classic MAC-like ductal proliferation occurring in MALTA, perineural invasion is absent, myoepithelial architecture is preserved, depth extends to the deep dermis, elastic fibers show pathognomonic papillary dermal clumping, architecture displays syringoma-like infiltrative ducts, and genetics demonstrate autosomal dominant *MYH9* pathogenic variants. In MALTA-BDSDP (this study), perineural invasion is absent, myoepithelial cells are preserved, depth extends unusually into deep subcutaneous tissue, elastic fibers show papillary dermal clumping, architecture reveals deep syringoid-like ducts with prominent apocrine features, and genetics demonstrate compound heterozygous *MYH9* pathogenic variants with autosomal recessive inheritance.

When these diagnostic features suggest MALTA rather than MAC, particularly bilateral or multifocal presentation in patients aged <40 years with a family history of similar lesions, germline *MYH9* sequencing of all 40 coding exons should be considered before committing to a definitive surgical intervention. Genetic testing may reveal autosomal dominant (single variant) or autosomal recessive (compound variants) inheritance patterns. This integrated approach stratifies patients into appropriate management: aggressive treatment with wide excision, Mohs surgery, and surveillance for MAC or conservative clinical monitoring, genetic counseling, and family screening for MALTA syndrome, thereby preventing misdiagnosis and unnecessary surgery while enabling appropriate genetic counseling.

The autosomal recessive inheritance pattern fundamentally challenges the current understanding of MALTA genetics. All previous cases have shown dominant inheritance, with single heterozygous variants producing the disease (Fewings et al, 2019; Fostier et al, 2023; Pecci et al, 2018). The family described in this study exhibited compound heterozygosity, with Gly455Ser and Ile1626Val in cis and Arg1497Gln in trans. Both heterozygous parents remained asymptomatic, and 1 compound heterozygous sibling showed no manifestations, demonstrating incomplete penetrance. This finding indicates that MALTA encompasses broader genetic heterogeneity than is currently recognized, with phenotypic expression modulated by additional factors. For genetic counseling, single-variant carriers may not develop the disease, whereas compound heterozygotes show variable expressivity, requiring individualized assessment.

Molecular modeling provides mechanistic insights. Myosin-9’s motor domain mediates adenosine triphosphate hydrolysis and actin binding; the tail domain enables filament assembly through coiled–coil dimerization. Different domain variants produce distinct phenotypes. MATINS-associated pathogenic variants are distributed along both motor and tail domains of *MYH9*, causing systemic manifestations (thrombocytopenia, nephropathy, and hearing loss) (Fewings et al, 2019; Shen et al, 2024), whereas pathogenic variants associated with MALTA affect cutaneous elastic fiber homeostasis in a tissue-restricted manner (Fostier et al, 2023; Pecci et al, 2018). The Gly455Ser motor sequence variant disrupts local packing, whereas Ile1626Val destabilizes coiled–coil hydrophobic interactions. Homodimer modeling showed that these domain-specific perturbations synergistically disrupt both adenosine triphosphate–dependent function and filament assembly (combined ΔΔG = −0.43 kcal/mol). Single-domain variants may be partially compensated, whereas multidomain disruption exceeds the functional threshold, manifesting as impaired elastic fiber homeostasis and BDSDP (Zhu et al, 1993).

To our knowledge, the 2 previously unreported variants (Gly455Ser and Arg1497Gln) expand MALTA's mutational spectrum. Gly455Ser received a high AlphaMissense pathogenicity score (0.997), whereas Arg1497Gln received a low score (0.126). Both variants segregated across 2 generations in a pattern consistent with autosomal recessive inheritance. Autosomal recessive MALTA may be underrecognized because asymptomatic heterozygous carriers do not prompt genetic investigation. As genomic sequencing becomes routine, systematic *MYH9* evaluation in MAC-like lesions that lack perineural invasion may reveal additional recessive cases.

This single-family case has inherent limitations. The recessive pattern requires validation in additional families, and although structural predictions using established methods correlate with the phenotype, functional validation through in vitro studies would strengthen the conclusions. The asymptomatic sibling suggests that additional modifiers exist. Despite these limitations, convergent evidence from family segregation, structural modeling, and well-defined histopathology compellingly supports the reported variants and inheritance pattern.

In conclusion, this case establishes BDSDP as a distinct histopathologic subtype of MALTA, expands the genetic spectrum of the syndrome to include autosomal recessive inheritance, and provides mechanistic insights into synergistic domain-specific effects on Myosin-9 function. The integration of clinical presentation, histopathology with elastic fiber staining, and targeted genetic testing will be essential for accurate diagnosis and genetic counseling as additional cases are identified. These observations provide a foundation for the future development of formal diagnostic criteria, which will require validation in additional families. Clinicians evaluating MAC-like lesions in young patients with bilateral presentation should consider *MYH9* testing when features suggest benign disease, recognizing that both dominant and recessive inheritance patterns may occur.

## Materials and Methods

### Tissue collection and histopathological evaluation

Biopsy samples were collected from the left temple, right cheek, and right nostril after consultation with a board-certified dermatopathologist (DJG). Tissues were fixed in 10% neutral buffered formalin, paraffin embedded, and processed for genomic and ancillary immunohistochemical analyses. Initial histopathologic evaluation was performed on 3-μm sections stained with H&E. Immunohistochemistry was performed on formalin-fixed, paraffin-embedded tissue sections using antibodies against GCDFP-15, EMA, and CD15 to assess apocrine differentiation and apical decapitation secretion. In addition, p63 immunohistochemistry stained the nuclei of basal myoepithelial cells lining the outer wall of the ductal structures. The left temple biopsy material was used for genomic analysis, whereas an angiofibroma from the right nostril served as a control.

### Genomic DNA extraction and sequencing

For genomic studies, 10-μm sections were cut from each paraffin block, and saliva samples were collected from the patient and relatives using Disposable Sampler Saliva Collection Kits (Wuxi NEST Biotechnology, Wuxi, China). Genomic DNA was extracted from formalin-fixed, paraffin-embedded samples from the zygomatic arch lesion and from saliva to confirm the presence of germline pathogenic variants using the QIAamp DNA FFPE Advanced kit (QIAGEN, Hilden, Germany) and the Monarch Spin gDNA Extraction kit (New England Biolabs, Ipswich, MA), respectively. Amplification of all 41 exons from *MYH9* was performed using genomic DNA as a template and the Power SYBR Green kit (Applied Biosystems, Thermo Fisher Scientific, Waltham, MA), with the following cycling program: 95 °C for 10 minutes; 45 cycles of 95 °C for 15 seconds, 58 °C for 20 seconds, and 60 °C for 75 seconds; followed by 65 °C for 2 minutes and a melt curve from 65 to 95 °C. Exons of interest were also amplified using Q5-HF 2x Mastermix (New England Biolabs). Primers used for amplification included those reported by Kunishima et al (2001), along with additional primers (Table 2) designed using the PrimerQuest Tool (Integrated DNA Technologies, San Diego, CA). Amplicons were treated with ExoSAP-IT PCR Product Cleanup Reagent (Applied Biosystems, Foster City, CA) according to the manufacturer’s instructions and submitted to Eurofins Genomics (Louisville, KY) for Sanger sequencing. The resulting sequence data were analyzed using Sequencher (version 5.4.6) software (Gene Codes, Ann Arbor, MI). Identified variants were evaluated for frequency and clinical relevance using VarSome (Saphetor, Lausanne, Switzerland) (Kopanos et al, 2019).

**Table 2:** Primers Used for the Amplification of the *MYH9* Gene

Primer	Sequence (5′–3′)
MYH9-4 reverse	CCTCAAGAATGAGAACAGACTGG
MYH9-9 reverse	GGAATCATTTTCCCATACACTGAAG
MYH9-12 forward	GGGCATAGGGTATGAGGGTTT
MYH9-12 reverse	CCACACCCAACCAAAGTCTTCA
MYH9-28 forward	TGGATCTAGGGTCCAGTGATGA
MYH9-28 reverse	GCCAGTTTGAGAAGAGAGAGAGAC
MYH9-29 forward	TTCTCAAACTGGCTCCTCAGAC
MYH9-29 reverse	GGAGCTGGTCCTGCTGATTTA
MYH9-32 forward	ATGCACGGGACTGTGTGTATT
MYH9-32 reverse	TTCCAGCTGCGTCTTCATCTC
MYH9-33 forward	ACGGAGATGGAGGACCTTATGA
MYH9-33 reverse	CAATCCAGGTGGAAGGAGAGAAC
MYH9-36 forward	CTAGAGGGTTTCTGGAGGAAGG
MYH9-36 reverse	CGTTGATCAGCTCCGTGTTG
MYH9-37 forward	CCCTGGCGTTAGAGGAGAAG
MYH9-37 reverse	CTTCTGAACACCCAACACAGAAG
MYH9-38 forward	TGGCTTCTGTGTTGGGTGTT
MYH9-38 reverse	CCAACCTGTGGAAGGGATGAG
MYH9-39 forward	TAAGAAGCCGGGTACACATAGG
MYH9-39 reverse	CTGCTTCAGGCGGGTAGAT
MYH9-40 forward	GAACGCCGAGCAGTACAAG
MYH9-40 reverse	CTCTGGTTGAGGAACAAGCTAC

### Model prediction

The Myosin-9 structure was predicted using AlphaFold3 (Jumper et al, 2021) and retrieved through UniProt (accession number P35579). The resulting model (AF-P35579-F1) showed high confidence scores across most of the sequence, with per-residue predicted Local Distance Difference Test values above 90, indicating a highly reliable structural prediction. Accordingly, the highest-confidence regions were primarily located in protein–protein binding domains and well-structured coiled–coil regions, consistent with high-resolution crystallographic data (PDB identifiers 3ZWH, 4CFQ, 4CFR, and 4ETO). Three short disordered regions—residues 1035–1057, 1118–1137, and 1877–1960—were identified on the basis of lower per-residue predicted Local Distance Difference Test scores. All structural visualizations and graphs were generated using PyMOL (The PyMOL Molecular Graphics System, version 2.5, Schrödinger, LLC, New York, NY).

### AlphaMissense pathogenicity prediction for *MYH9* variants in Myosin-9

Missense mutations in Myosin-9, including Ser232Asn, Gly236Cys, Gly455Ser, Lys651Thr, Cys671Tyr, Arg1497Gln, and Ile1626Val, were evaluated using the AlphaMissense Pathogenicity Heatmap, which integrates AlphaFold structural data with evolutionary and functional features (Cheng et al, 2023). Each substitution is scored from 0 (benign; blue in the heatmap) to 1 (highly pathogenic; red in the heatmap). Motor domain variants Ser232Asn, Gly236Cys, Gly455Ser, Lys651Thr, and Cys671Tyr received high pathogenicity scores (0.999, 0.998, 0.997, 0.983, and 1.000, respectively), whereas tail domain variants Arg1497Gln (0.126) and Ile1626Val (0.086) received low pathogenicity scores, suggesting domain-specific effects on protein function.

### Modeling of Myosin-9 dimers

Homodimeric models of human wild-type (UniProt: P35579, https://www.uniprot.org/uniprotkb/P35579/entry) and mutant Myosin-9 were generated using AlphaFold-Multimer (version 2.3.2) (Homma et al, 2023; Omidi et al, 2024). Amber minimization was applied to the wild-type model but not to the Gly455Ser-Ile1626Val:Arg1497Gln (also [G455S-I1626V]:R1497Q) variant model owing to computational resource constraints. Structural evaluation was performed using SWISS-Model to evaluate stereochemistry and to estimate global and local model quality of the top models ranked by AlphaFold3 (Studer et al, 2020). These models were further analyzed using the Schrödinger Suite (Schrödinger Release 2025-2: Prime, Schrödinger, LLC). Residue scanning was performed on the wild-type Myosin-9 homodimer to determine changes in free energy (ΔΔG) associated with the G455S, I1626V, and R1497Q pathogenic variants, focusing on their impact on homodimer stability and interchain binding affinity. The variants were modeled simultaneously, with G455S and I1626V occurring on Chain A and R1497Q on chain B (Figure 5a). Changes in structural stability (*ΔΔG*stability) were calculated as the difference in free energy between the unfolded and folded states of the wild-type and variant dimers (Figure 5b). Changes in binding affinity (*ΔΔG*bind) between dimer chains were computed as the difference in free energy change between the wild-type and mutant homodimer monomers (Figure 5b). Both *ΔΔG*stability and *ΔΔG*bind were estimated using Schrödinger Prime MM-GBSA (Jacobson et al, 2004, 2002). Changes in hydropathy for each sequence variant substitution were reported on the basis of the Kyte–Doolittle scale to indicate shifts in hydrophobicity or hydrophilicity (Kyte and Doolittle, 1982) (Figure 5b). Model quality metrics indicate moderate confidence (Figure 4), with Ramachandran plots supporting the predicted secondary structure (Figure 4e and k). Although deviations from ideal geometry and energetic QMEAN scores are present (Figure 4b and h), the models fall within the quality range observed in high-resolution experimental structures (Figure 4d and j).

### In silico analysis of protein stability

DynaMut2 was used to assess the structural and thermodynamic effects of missense sequence variants in Myosin-9 (Rodrigues et al, 2021). This tool combines normal mode analysis and graph-based signatures to evaluate how single-point sequence variants influence protein stability and conformational flexibility. For each variant, DynaMut2 predicts the change in Gibbs free energy (*ΔΔG*, in kcal/mol) upon each sequence variant. Variants yielding *ΔΔG* values < 0 were interpreted as destabilizing (ie, leading to reduced protein stability), whereas those with *ΔΔG* > 0 were classified as stabilizing. Structural analysis was performed using the AlphaFold-predicted Myosin-9 (UniProt P35579) as input. Outputs included both *ΔΔG* values and graphical representations of altered residue interactions, including hydrogen bonds, Van der Waals forces, and electrostatic contacts, comparing wild-type with variant structures.

## Ethics Statement

All research was conducted according to the ethical standards set forth by the Virginia Tech Carilion and the Fralin Biomedical Research Institute Institutional Review Board committees (IRB 25-1203); written, informed consent was obtained from the subjects of this study. The patient also provided written informed consent for the use of samples and images in research studies and publication.

## Data Availability Statement

All data generated or analyzed during this study are included in this article and its figures and are available from the corresponding author upon reasonable request. Genetic variants identified in MYH9 have been deposited in the DNA Databank of Japan (https://ddbj.nig.ac.jp/search/entry/bioproject/PRJDB42609/). Raw sequencing chromatograms, in silico modeling files, and full-resolution histopathology images can be shared upon request in accordance with institutional and ethical guidelines. Structural modeling was performed using publicly available tools (AlphaFold, AlphaMissense, and DynaMut2); detailed parameters are provided in Materials and Methods.

## ORCIDs

Carla V. Finkielstein: http://orcid.org/0000-0002-8417-4643

Douglas J. Grider: http://orcid.org/0000-0002-6346-094X

Etta Hanlon: http://orcid.org/0009-0000-7489-4981

Daniel G. S. Capelluto: http://orcid.org/0000-0002-0412-4508

Matthew G. Urbano: http://orcid.org/0009-0005-2252-7860

Katherine L. Brown: http://orcid.org/0000-0002-6514-8976

Haley M. Michel: http://orcid.org/0000-0002-4787-3410

Clinton Roby: http://orcid.org/0000-0002-0363-6712

Dzenis Mahmutovic: http://orcid.org/0000-0002-2180-2147

Anne M. Brown: http://orcid.org/0000-0001-6951-8228

Priyenka Khatiwada: http://orcid.org/0009-0002-2906-3219

## Conflict of Interest

DJG served on an advisory board for Castle Biosciences regarding melanoma prognostic testing. AMB holds shares in Beam Diagnostics, which is unrelated to this work. The remaining authors state no conflict of interest.
